# Navigation-Assisted One-Stage Total Knee Arthroplasty With Extra-Articular Corrective Osteotomy for Knee Osteoarthritis With Femoral and Tibial Extra-Articular Deformity: A Case Report

**DOI:** 10.1155/2024/6699418

**Published:** 2024-07-25

**Authors:** Mitsuhiko Kubo, Sho Hirobe, Tsutomu Maeda, Kosuke Kumagai, Yasutaka Amano, Yuki Nosaka, Takahide Hasegawa, Shinji Imai

**Affiliations:** ^1^ Department of Sports and Musculoskeletal Medicine Shiga University of Medical Science, Seta, Tsukinowa-cho, Otsu, Shiga 520-2192, Japan; ^2^ Department of Orthopaedic Surgery Shiga University of Medical Science, Seta, Tsukinowa-cho, Otsu, Shiga 520-2192, Japan

## Abstract

**Background:** Knee osteoarthritis (OA) with extra-articular deformity (EAD) is a rare condition for which achieving accurate alignment with total knee arthroplasty (TKA) is difficult. Extra-articular corrective osteotomy may be necessary for severe deformities.

**Case Presentation:** A 76-year-old man underwent TKA for knee OA with EAD due to malunion after fractures of the femur and tibia. The femoral varus and the tibial valgus/recurvatum deformities were mild and corrected by intra-articular osteotomy using navigation (i.e., navigation-assisted standard TKA). However, the femoral antecurvatum deformity was severe, and we performed extra-articular corrective osteotomy simultaneously with TKA. Navigation was used not only for TKA but also for extra-articular corrective osteotomies. The osteotomy site was fixed with a cemented stem and metaphyseal sleeve. The postoperative hip–knee–ankle angle was 1° varus, the femoral implant was implanted at 0.5° varus/0.5° flexion, and the tibial implant was implanted at 0.5° varus/0° posterior slope. Two years after surgery, improvements were obtained in the range of motion from 15°–95° to 0°–110°, the Knee Society Score from 39 to 92 points, and the functional score from 35 to 100 points.

**Conclusions:** One-stage TKA with extra-articular corrective osteotomy achieved good clinical results due to accurate alignment using navigation and firm fixation of the osteotomy site using cemented-stem and metaphyseal sleeve without any fixation devices.

## 1. Introduction

In total knee arthroplasty (TKA), the lower limb and implant alignment affect the implant survival and knee joint function. Knee osteoarthritis (OA) with extra-articular deformity (EAD) is a rare condition for which achieving accurate alignment with TKA is difficult. If the deformity is mild, TKA with an intra-articular corrective osteotomy (i.e., standard TKA) can correct the deformity [1–3]. In recent years, good results for TKA with an intra-articular corrective osteotomy using navigation have been reported [4, 5]. However, in case of severe deformity, an extra-articular corrective osteotomy may be necessary to achieve accurate alignment [6–15]. There are two types of procedures for an extra-articular corrective osteotomy: a two-stage procedure in which corrective osteotomy is performed first, followed by TKA, and a one-stage procedure, in which corrective osteotomy and TKA are performed simultaneously [6, 7]. The two-stage procedure requires two surgeries, resulting in a longer treatment period. Therefore, the one-stage procedure is theoretically advantageous. However, the one-stage procedure is more invasive, especially in cases where the osteotomy site is far from the operation field of TKA, because two incisions are required. Therefore, the best indication of the one-stage procedure is in cases whereby only one incision site is needed to perform the two operations. On the other hand, because the most common complication is nonunion at the osteotomy site, the cases in which firm fixation is impossible due to osteoporosis are the indication for the two-stage procedure. So far, there are relatively few reports of the one-stage procedure due to the difficulty of the procedure [6–15].

Here, we describe a patient treated with navigation-assisted one-stage TKA with extra-articular corrective osteotomy for knee OA with EAD due to malunion after femoral and tibial fractures. The femoral varus and tibial valgus/recurvatum deformity was mild and corrected by intra-articular osteotomy using navigation (i.e., navigation-assisted standard TKA). However, the femoral antecurvatum deformity was severe, and we performed extra-articular corrective osteotomy simultaneously with TKA. We used navigation not only for TKA but also for extra-articular corrective osteotomy. To the best of our knowledge, this is the first report of navigation-assisted one-stage TKA with extra-articular corrective osteotomy.

## 2. Case Presentation

### 2.1. Patient Background

**Figure 1:**
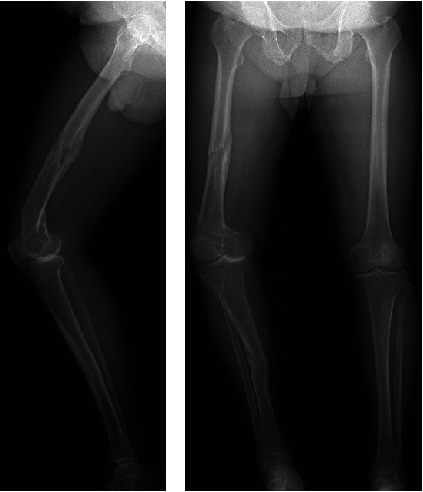
Preoperative radiograph. (a) Lateral view: Femoral deformity is 25° antecurvatum; tibial deformity is 7° recurvatum. (b) Anteroposterior view: Hip–knee–ankle angle is 21.7° varus, femoral deformity is 7° valgus, and tibial deformity is 7° valgus.

A 76-year-old male patient presented to our hospital with right knee joint pain. He was a horse trainer and had a history of two femoral fractures and one tibial fracture. Femoral and tibial shaft fractures were treated operatively; however, femoral supracondylar fracture was conservatively treated with cast. Femorotibial OA was radiographically classified as Kellgren–Lawrence Grade IV. Due to postfracture malunion, the overall alignment of the lower limb revealed a hip–knee–ankle angle of 21.7° varus, the femoral deformity was 7° varus in the coronal plane/25° antecurvatum in the sagittal plane, and tibial deformity was 7° valgus in the coronal plane/7° recurvatum in the sagittal plane (Figure 1).

### 2.2. Preoperative Planning

**Figure 2:**
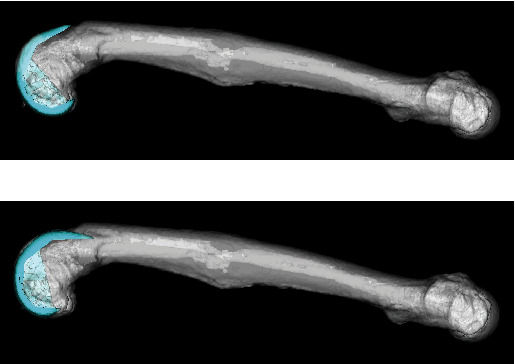
Preoperative planning using a 3D digital template. (a) The femoral implant is placed in line with the distal femoral axis. (b) The femoral implant is placed in line with the femoral mechanical axis.

Preoperative planning was performed using a 3D digital template (ZedKnee, LEXI, Tokyo, Japan). Femoral varus and tibial valgus/recurvatum deformity could be corrected by intra-articular corrective osteotomy, in which the articular surfaces of both femur and tibia were osteotomized perpendicular to the mechanical axis. Regarding the sagittal alignment of femoral component, when the femoral implant was placed in line with the distal femoral axis in the sagittal plane, an extension deficit of 25° could develop (Figure 2(a)). When the femoral implant was placed in line with the femoral mechanical axis in the sagittal plane, a notch was observed (Figure 2(b)). From these simulations of the operative procedure, we considered that extra-articular corrective osteotomy was necessary for the femoral antecurvatum deformity. We planned a one-stage TKA with extra-articular corrective osteotomy by 25° extension at the deformity apex of femur.

### 2.3. Operation and Postoperative Rehabilitation

**Figure 3:**
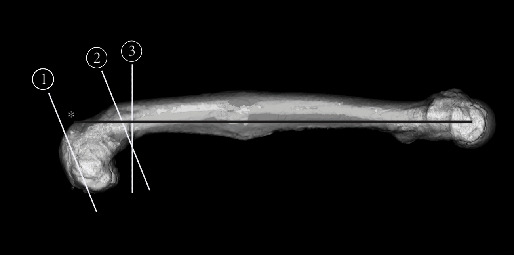
Osteotomy lines. Line 1: Distal femur osteotomy has been performed to fix the varus/valgus to a neutral position with 25° of flexion. Lines 2 and 3: A 25° closed-wedge extension corrective osteotomy. Gray line: mechanical axis on the sagittal plane after corrective osteotomy. ^∗^Distal femoral reference point.

**Figure 4:**
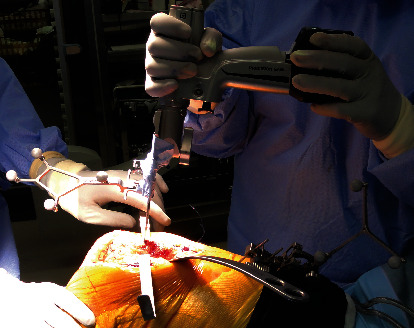
Extra-articular osteotomy using navigation. Extra-articular corrective osteotomy was performed at the deformity apex using the Stryker Precision™ Oscillating Tip Saw (Stryker, New Jersey, United States) mounted with a navigation tracker.

We performed TKA using the Attune™ knee system (DePuy, Warsaw, Indiana, United States) using CT-free navigation (Kolibri, BrainLAB). The tibial valgus and recurvatum deformity were corrected by intra-articular osteotomy (i.e., navigation-assisted standard TKA), aiming for a neutral position of the varus/valgus and 0° posterior slope. Navigation was also applied for the femoral extra-articular corrective osteotomy. The femoral tracker was placed proximal to the osteotomy site so that navigation could be used for both TKA and extra-articular corrective osteotomies. During the registration process of the navigation system, we registered the distal femoral reference point as the distal endpoint of the expected mechanical axis in the sagittal plane after corrective osteotomy to enable navigation use after corrective osteotomy (Figure 3). First, navigation-assisted distal femur osteotomy was performed with the aim of achieving a neutral position of the varus/valgus and 25° of flexion (Figure 3, Line 1). Subsequently, a 25° navigation-assisted closed-wedge extension corrective osteotomy was performed at the apex of deformity. We used navigation to decide the coronal and sagittal alignment of the osteotomy line in EAD. The coronal alignment was aimed perpendicular to the mechanical axis, the sagittal alignment of the first osteotomy line was aimed at 25° flexion, and that of the second osteotomy line was aimed perpendicular to the mechanical axis. The amount of bone resection was determined by the point at which the two osteotomy lines were crossed, just at the posterior cortex of femur (Figure 3, Lines 2 and 3). The Stryker Precision™ Oscillating Tip Saw (Stryker, New Jersey, United States) was mounted with the tracker of navigation (Figure 4), and the osteotomy was performed without a cutting guide. We corrected the rotational deformity of the femur by temporarily fixing the osteotomy site with a stemmed femoral osteotomy guide and rotating the distal fragment freely depending on the surrounding soft tissue. The rotational alignment of the femoral implant was determined using the gap balancing technique, while that of the tibial implant was determined by the range-of-motion technique [16, 17]. The femoral osteotomy site was fixed with the cemented stem and the metaphyseal sleeve. Postoperatively, the patient was immobilized with a knee brace for 1 week, and partial weight-bearing was started at 4 weeks; full weight-bearing was allowed at 8 weeks.

### 2.4. Radiological and Clinical Results

**Figure 5:**
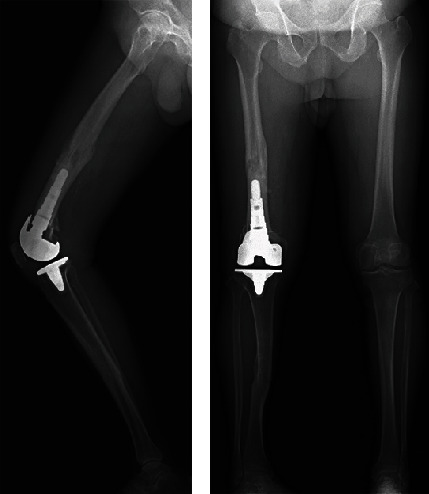
Postoperative radiograph. (a) Lateral view: Femoral and tibial implants are implanted at 0.5° varus. (b) Anteroposterior view: Hip–knee–ankle angle is 1° varus, the femoral implant is implanted at 0.5° flexion, and the tibial implant is implanted at 0° posterior slope.

The postoperative hip–knee–ankle angle was 1° varus, the femoral implant was implanted at 0.5° varus/0.5° flexion, and the tibial implant was implanted at 0.5° varus/0° posterior slope (Figure 5). Two years after surgery, the range of motion improved from 15°–95° to 0°–110°, the Knee Society Score improved from 39 to 92 points, and the functional score improved from 35 to 100 points.

## 3. Discussion

We performed navigation-assisted one-stage TKA with extra-articular corrective osteotomy for knee OA with EAD due to malunion after femoral and tibial fractures. The femoral varus and tibial valgus/recurvatum deformities were mild and were corrected by intra-articular corrective osteotomy (i.e., standard TKA). However, the femoral antecurvatum deformity was severe and was corrected by extra-articular corrective osteotomy. We used navigation not only for TKA but also for extra-articular corrective osteotomy. The femoral osteotomy site was firmly fixed with the cemented stem and the metaphyseal sleeve without any fixation devices.

The indication for femoral extra-articular corrective osteotomy is controversial. For cases of coronal deformity, EAD can be corrected by intra-articular corrective osteotomy (i.e., standard TKA) if the collateral ligament attachment is not damaged by the perpendicular osteotomy for the mechanical axis [1]. In general, extra-articular osteotomy is recommended for EAD exceeding 15° [1, 18, 19]. For cases of recurvatum deformity, it is possible to correct the alignment by placing the implant in a flexed position relative to the distal bone axis. However, in cases of antecurvatum deformity, it is not possible to place the implant in an extended position relative to the distal bone axis due to the risk of notching [8]. Therefore, we performed extra-articular corrective osteotomy.

Whether it is better to perform osteotomy and TKA in one or two stages is also controversial [18]. The one-stage procedure is a technically demanding surgery with an increased risk of nonunion at the osteotomy site. However, it has the advantage of requiring only one operation, resulting in a shorter treatment period [9, 20]. Therefore, if there is a method that allows accurate osteotomy and firm fixation of the osteotomy site, a one-stage osteotomy would be preferable for the patient.

Navigation is an effective tool to obtain an accurate alignment in regular TKA. Navigation has also been reported to be effective for TKA in the cases of knee OA with EAD [4, 5]. We were able to correct the femoral varus and tibial valgus/recurvatum deformity with TKA using navigation. Moreover, we were able to correct femoral antecurvatum deformity with extra-articular corrective osteotomy using navigation. This is the first report of a navigation-assisted one-stage TKA with extra-articular corrective osteotomy.

Extra-articular corrective osteotomy is usually performed using the freehand technique. This is one of the reasons for the complexity of this procedure. As a device to correct osteotomy in one-stage TKA with extra-articular osteotomy, a triangular “guide” made preoperatively to match the corrective angle was reported to confirm the osteotomy angle [21]. There are also reports of the use of the patient-specific instrument (PSI) [10]. Shao et al. reported good results using the PSI for one-stage TKA with extra-articular corrective osteotomy in patients with malunion after femoral fracture [10]. In the present case, we performed extra-articular corrective osteotomy using navigation.

Another major challenge is fixation of the osteotomy site. Various fixation methods such as plate and screw [6], nail [11], and stem extension [12] have been reported. If additional fixation devices are used, hardware removal [21] and manipulation due to poor range of motion have previously been reported in some cases [6, 8, 21]. The stem extension can be simply added to the TKA implant and is easy to use, and good results had been reported with fixation of the stem extension [21]. However, fixation with a stem extension alone may not be sufficient [12, 13], and there are reports stating the need for additional plate fixation [8, 9, 20]. Some reports recommend the use of long stem extensions [20, 21] or cement fixation of the stem extension to increase the fixation strength [14]. In this case, we cemented the stem extension and additionally applied a metaphyseal sleeve. There have been no previous reports of one-stage TKA with extra-articular corrective osteotomy wherein the osteotomy site was fixed with a cemented stem and metaphyseal sleeve.

The limitation of this surgery is that it can only be performed in cases of knee OA with EAD due to malunion of supracondylar femoral fracture. The incidence of supracondylar femoral fractures is 8.7/100,000/year [22]; although not a frequent fracture, its incidence increases with age [22]. Also, according to a recent meta-analysis, the incidence of malunion of the supracondylar femoral fracture is 18.3% [23], which is quite high. As the population ages, patients with knee OA with malunion of the supracondylar femoral fracture are expected to increase further. Therefore, this operation can be a useful treatment option.

## 4. Conclusion

Herein, we report a case of knee OA with EAD due to malunion after femoral and tibial fractures treated with one-stage TKA with extra-articular corrective osteotomy. The femoral antecurvatum deformity was severe and corrected with simultaneous extra-articular corrective osteotomy with TKA. Navigation was used for extra-articular corrective osteotomies in addition to TKA. The femoral osteotomy site was firmly fixed with a cemented stem and metaphyseal sleeve. Navigation-assisted one-stage TKA with extra-articular corrective osteotomy can be a useful treatment option.

## Data Availability

The authors have nothing to report.
